# Impaired fasting glucose, oxidative distress, and cognitive impairment. Is this the starting point on DBT cognitive decline?

**DOI:** 10.3389/fnagi.2022.911331

**Published:** 2022-07-26

**Authors:** María Pilar Canal, Karen Agustina Nini, Maria Verónica Baez

**Affiliations:** ^1^Instituto de Biología Celular y Neurociencia “Prof. E. De Robertis” (IBCN, CONICET-UBA), Buenos Aires, Argentina; ^2^1°UA de Histologia, Embriología, Biologia Celular y Genética, Facultad de Medicina, Universidad de Buenos Aires (UBA), Buenos Aires, Argentina

**Keywords:** impaired fasting glucose, redox imbalance, cognitive decline, cross-sectional studies, DBT2

## Abstract

Different studies performed in human patients, animal models, and *in vitro* cell cultures, show a correlation between type 2 diabetes (DBT2) and certain neurodegenerative pathologies. Also, it was proposed that increased inflammation and- or oxidative distress are a possible cause of DBT2-accelerated cognitive decline. The onset of DBT2 is characterized by an increase in blood glucose levels due to (an inability of the body’s cells to use insulin properly) called impaired fasting glucose (IFG). Genetic and/or molecular causes of IFG have not yet been established, but metabolic syndrome, obesity, unbalanced diets, and sedentary lifestyle would be responsible, at least in part, for the multiplication in the number of this disease. It has been proposed that hyperglycemia itself causes an imbalance in the redox state and could compromise blood-brain barrier (BBB) causing neurodegeneration. For this reason, we propose, in this review, to evaluate the available data about redox state and neurocognitive studies during the IFG period.

## Introduction

Type 2 Diabetes (DBT2) is a metabolic/systemic type syndrome characterized by the deregulation of carbohydrate, lipid, and protein metabolisms, which affects the whole organism and has in the nervous system one of its main targets (Carvalho et al., 2015; DeFronzo et al., 2015; Tokarz et al., 2018). DBT2 and neurodegeneration are closely related (Roriz-Filho et al., 2009; Yau et al., 2010; Ahmed et al., 2014; Butterfield et al., 2014; Carvalho et al., 2015). Both hyperglycemia (HG), by itself, and-or combined with hyperinsulinemia, have been associated with an increased rate of age-related cognitive decline (Zhou et al., 2015), vascular dementia (Carvalho et al., 2013), and Alzheimer’s disease (Carvalho et al., 2013, 2015; Butterfield et al., 2014). High glucose levels (observed in DBT2 and/or poor glycemia control) have been shown to induce neurodegeneration *via* advanced glycation end products (AGEs), reactive oxygen species (ROS), and inflammation (Athanasaki et al., 2022). Potenza et al. reviewed that AD could be started in DBT2 patients due to a decline in mitochondrial function that would increase ROS circulating levels causing elevated tau expression/phosphorylation and Aβ accumulation, synapse loss, impaired learning, and memory; and synaptic plasticity deficits (Potenza et al., 2021). More recently, Sanotra and collaborators, showed that chronic HG is highly associated with lipid peroxidation [measured as 4-hydroxynonenal (HNE) levels], general cognitive decline and neurodegeneration. Also, they observed that AD patients show high blood glucose levels and elevated HNE levels in the brain and blood. The authors conclude that HG increases oxidative distress levels which in turn, induces lipid peroxidation products, such as HNE, that contribute to lower antioxidant defense mechanisms and lead to neurodegeneration (Renuka Sanotra et al., 2022).

HG is the first sign in DBT2 development. Impaired fasting glucose (IFG) is considered a risk factor for DBT2, as well as a first step in the development of this disease. IFG is characterized by a slight increase in glycemia (Glu < 126 mg/dl or 6.99 mM) with normal insulin levels. During this stage, patients do not show any signs of disease. However, the increase in glucose levels can lead to severe consequences including an increase in protein glycation, endothelium damage, and inflammatory markers levels, in a way that is not evident until DBT2 is established.

Until 2006, The World Health Organization (WHO) considered that glycemic values ranging between 110 and 126 mg/dl (6.11–6.99 mM) correspond to IFG (WHO, 2006). However, the American Diabetes Association (ADA) considered IFG to be present when glucose values were higher than 100 mg/dl (5.55–6.99 mM). Furthermore, the ADA criteria includes impairment in glucose tolerance (IGT), altered glycemia values obtained after 1 or 2 h in an oral glucose tolerance test (OGTT) and/or HbA1c between 5.7% and 6.4% as evidence of IFG (American Diabetes Association, 2019). These results must be confirmed in a second test, which represented a challenge in treating patients with lower economic status, and in lower income countries. Furthermore, IFG patients could have glucose levels slightly and constantly increased (which is detectable) or alternate between hyperglycemia peaks and normal fasting glucose levels (which may be difficult to detect in routine laboratory practice). Altogether, these facts led to underdiagnosis of IFG. Since 2022, the American Diabetes Association defines the term “prediabetes” the individuals whose glucose levels do not meet the criteria for diabetes yet have abnormal carbohydrate metabolism (American Diabetes Association Professional Practice Committee, 2022).

In 2015, Koekkoek et al. (2015) affirmed that the risk of dementia was increased in the prediabetic stages since prediabetes was associated with an increased incidence of dementia. However, this association was confirmed only when Insulin Resistance (IR) is also observed (Willette et al., 2013). Considering that chronic hyperglycemia could develop neurodegeneration and possibly AD by oxidative distress mechanisms, and that there is still controversy that the IFG stage (before IR development) could be associated with this phenomenon, we review in this article the data associated with both: IFG and cognitive impairment and IFG and redox state, looking for a relationship between these three parameters.

## IFG and Redox State

It is well known that, in DBT2 and IR, hyperglycemia induces protein glycation, both in erythrocytes and in endothelial proteins. This leads to: (1) an increment in the permeability of the renal filtration barrier at the kidneys and the blood-brain barrier (BBB) at the central nervous system (CNS); and (2) an increase in the rigidity of the capillary endothelium that results in a rise in blood pressure. In parallel, glycation of erythrocytes decreases their viability and leads to an increase in HbA1c %, which is a marker of DBT2 at all disease stages (DeFronzo et al., 2015). Higher glucose levels also induce an increase in advanced glycation end products (AGEs), ROS, and mitochondrial DNA as a result of a higher oxidative state, which can be detected in the sera of patients with DBT2 (Rodríguez-Ramírez et al., 2017; La Sala et al., 2019). However, the redox state during IFG has been poorly investigated.

In humans, Rodríguez-Ramírez et al. (2017) observed that patients with IFG, as defined by the ADA criteria, showed significantly higher serum levels of malondialdehyde as well as lower total antioxidant capacity than those in the control group. Furthermore, La Sala et al. (2019) showed that elevated levels of micro-RNA-21 (miR-21) were associated with an increased abundance of ROS levels in sera from volunteers with IGT as determined by an OGTT. The authors also described a decrease in superoxide dismutase-2 (SOD2) levels in the sera of volunteers with IGT. More important, miR-21, ROS, and SOD2 levels found in the sera of volunteers with IGT were intermediate between those of normal volunteers and those of patients with DBT2 (La Sala et al., 2019). Additionally, Maschirow et al. (2015) found that patients with IFG presented an increase in erythrocyte oxidative distress, measured by a reduction in the reduced to oxidized glutathione (GSH/GSSG) ratio when compared to healthy individuals. These results indicate that IFG may lead to an alteration in redox state, and also that there may be a positive correlation between redox imbalance and DBT2 progression. However, in any case, the authors did not study the cognitive state in the analyzed population.

IFG is difficult to study on the bench. Murine models do not reproduce IFG/IGT status, as rodents’ genomes codify for two active insulin genes (insulin 1 and 2) that help in glucose homeostasis preventing hyperglycemia (Kakita et al., 1982). Thus, in IR and DBT2 animal models, insulin levels increase before glycemia does. As animal models are not available, several studies have attempted to mimic hyperglycemia *in vitro*, in cultured cells by adding glucose to the culture media. However, these studies are performed using high glucose concentrations, ranging from 10 to 33 mM (180–594 mg/dl; Kumar et al., 2017; Pal et al., 2020) which correspond to values observed in patients with advanced DBT2 or individuals with poor glycemic control (DeFronzo et al., 2015).

One of those that used the lowest range of glucose concentrations, Kumar et al. (2017), incubated primary astrocyte cultures and glioblastoma cell lines with 10 mM to 25 Mm glucose (180–450 mg/dl) and observed increased ROS levels and activation of Akt and ERK1/2. Furthermore, they found increased expression of proinflammatory markers such as TNF-α IL-1β, GFAP, VEGFα, and MMP-9, together with a decrease in BDNF, IL-6, and IL-10 (Kumar et al., 2017). More recently, Pal et al. (2020) observed an increase in ROS production in a retinal cell line (RGC-5), leading to the disruption of antioxidant defense mechanisms, following incubation in a high-glucose environment (by the addition of 20 mM glucose on top of that already present in DMEM supplemented with 10% FBS). In our laboratory, in a more physiological approach, we incubated primary mixed cultures of neurons and astrocytes with sera from patients with IFG for 1 h and observed an increase in stress granules similar to that obtained when neurons were treated with 100 μM H_2_O_2_ (Canal et al., in preparation).

In parallel, Rom et al. (2020) found that treating a primary culture of human brain microvascular endothelial cells (BMVEC) with AGEs resulted in a significant increase in ROS production. The authors also treated these cultures with 25 mM glucose and observed a reduction in trans-endothelial electrical resistance (TEER), which could translate to leakage in the BBB. This decrease in TEER was prevented when BMVEC cells were concomitantly exposed to glucose and a ROS inhibitor, indicating that the impairment in BBB permeability induced by hyperglycemia is likely mediated by ROS (Rom et al., 2020). Similarly, Liu et al. (2021) incubated human glomerular endothelial cells (HGECs) with high glucose concentrations (16.7 mM and 33.3 mM) and observed an increase in nitric oxide (NO) secretion compared to culture media without glucose supplementation. This increase was accompanied by increased expression of endothelial nitric oxide synthase (eNOS). Additionally, alterations in the levels of two molecular markers associated with an increase in oxidative distress were also detected: an increase in activated AKT (P-AKT), and a decrease in PTEN expression (Liu et al., 2021).

Altogether, these results indicate that under IFG conditions redox signaling is activated, coupled with increased ROS and stress signaling at the molecular level which in turn induce an increase in oxidative markers and a decrease in antioxidative ones that could be detected in patients’ samples. These alterations result in an intermediate redox state between IR-DBT2 and control statuses. The oxidative changes within the cells promote a proinflammatory status, thus priming the organism for progression to DBT2.

## IFG and Cognitive Impairment

**Figure 1:**
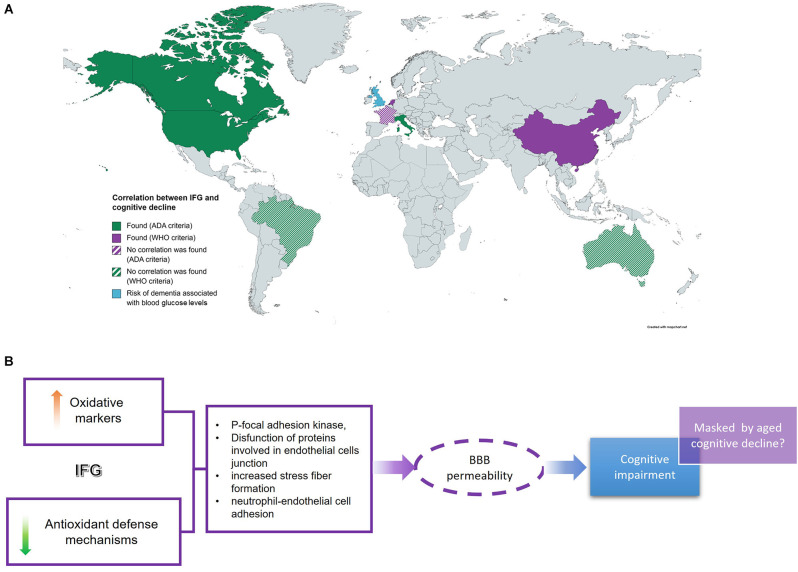
IFG and cognitive impairment. **(A)** Several cross-sectional studies around the world intended to find a correlation between IFG and cognition with different results. **(B)** IFG-related cognitive deficit is associated with an increase in oxidative markers and a decrease in redox defense systems which would affect endothelial cell permeability at BBB leading to neuro-damage and cognitive impairment. However, this is detectable at lower ages. IFG, impaired fasting glucose; BBB, blood-brain barrier.

Several demographic studies around the world have evaluated the cognitive state in different populations (Yaffe et al., 2004; Di Bonito et al., 2007; Rouch et al., 2012; Samaras et al., 2014; Cukierman-Yaffe et al., 2015; Hawkins et al., 2016; Zhang et al., 2016; Van Agtmaal et al., 2018; Xiu et al., 2019; Santos et al., 2021). However, these represent a small number of countries (Figure 1A); furthermore, the results reported by these studies are contradictory. On one hand, several reports describe some degree of cognitive impairment in volunteers with IFG (Yaffe et al., 2004; Samaras et al., 2014; Wright et al., 2015; Santos et al., 2021). However, some studies did not detect any significant differences between controls and volunteers with IFG (Cukierman-Yaffe et al., 2015; Santos et al., 2021). This controversy could be due to the different composition of volunteer populations in terms of age, socio-economical and/or educational status, as well as IFG duration. This last issue is difficult to assess as cross-sectional studies could not detect since when people recruited has this condition and, in several cases, insulin and or HbA1c levels are not measured, which could be indicative of hyperglycemic duration.

In a cross-sectional study conducted in healthy adults, Wright et al. (2015) discovered that higher levels of glucose under fasting conditions or after a 2-h OGTT were associated with better response inhibition and long-term verbal memory performance in participants over age 70, but not among participants below 70 years of age. The authors did not stratify volunteers into IFG or IGT groups, but the statistical analysis revealed that there were significant associations between fasting glucose, 2-h post-OGTT glucose levels, and neurocognitive function modified by age, sex, and education. In this study, poorer short-term verbal memory performance was observed among men and in individuals with lower educational levels (Wright et al., 2015). However, in Brazil, the Pietá Study (2021) did not reveal any significant differences in cognition between adult controls and volunteers with IFG (as per ADA criteria) of similar ages and lower educational levels (Santos et al., 2021). Similarly, the Sidney Memory and Ageing Study, performed on individuals between 70 and 90 years old, found that IFG (as per ADA criteria) was not associated with a decline in global cognition within 2 years of study (Samaras et al., 2014). However, the transition from normal glucose levels to IFG during the evaluation period was linked to an increase in cognitive decline.

On the other hand, Rouch et al. (2012) followed for 4 years, 163 non-diabetic participants (mean age 65) without dementia at recruitment and observed that the presence of DBT2 was associated with a larger decline in selective attention and executive functioning. However, this association was not observed between IFG (as per WHO criteria) and executive functioning or in episodic memory (Rouch et al., 2012). Similar results were obtained in the Multiple Outcomes of Raloxifene Evaluation (MORE) trial, which included healthy women and women with DBT2 or IFG (as per WHO criteria), mean age of 66.3 years, who were followed for 4 years. The results of the study showed that patients with DBT had worse performance on cognitive testing and displayed greater cognitive decline and risk of developing cognitive impairment compared to women without diabetes. In the same study, volunteers with IFG tended to have cognitive scores and rates of decline which were intermediate between those of healthy women and women with DBT (Yaffe et al., 2004).

Di Bonito et al. (2007) showed that high levels of fasting plasma glucose (above 110 mg/dl) correlated better with cognitive deficit than ADA criteria for IFG (>100 mg/dl) after administering the Mini-Mental State Exam (MMSE) test to 182 non-diabetic participants (between 65 and 78 years old). The authors also observed that insulin levels were powerful metabolic markers of impaired cognitive performance in elderly people (Di Bonito et al., 2007). This same evaluation was performed by Xiu et al. (2019) on a group of Chinese individuals over 55 years of age. In this study, the authors found that those participants classified as having IFG according to WHO criteria scored significantly lower in the MMSE test than the control group (Xiu et al., 2019).

Some studies have been performed on younger populations. Hawkins et al. (2016) investigated the role of higher fasting glucose values on obese young adults (around 20 years of age) and found that IFG (as per ADA criteria) was associated with poorer cognitive performance after adjusting for body mass index (BMI), particularly on tests of inhibitory control (Go/NoGo task; Hawkins et al., 2016). Similarly, Cukierman-Yaffe et al. (2015) followed a young population (starting at 17 years old) for 6 years and discovered that individuals with the lowest cognitive scores at the beginning of the testing had a two-fold greater risk for the development of IFG (as per ADA criteria) compared with those with the highest cognitive scores.

In parallel, a community-based cohort study showed that higher glucose levels were associated with an increased risk of dementia in populations without and with diabetes (Kielstein, 2013).

On the other hand, some studies chose to evaluate the link between IFG or DBT2 and brain damage from a structural point of view. Zhang et al. (2016) performed one such study in an Australian population (Men and women, mean age 63 years old). The authors found that elevated plasma glucose levels were associated with smaller regional volumes in the hippocampus and striatum (Zhang et al., 2016). Similarly, the Maastricht Study, performed in the Netherlands, evaluated patients with IFG (as per WHO criteria) with an average age of 60 years. In this study, the authors found an association between IFG and structural brain abnormalities, which constitute an increased risk for the development of dementia (Van Agtmaal et al., 2018).

Altogether, the results reviewed in this section show that IFG is associated with several brain abnormalities that could increase the risk for cognitive impairment. In fact, some studies identified cognitive deficits in patients with IFG that were more evident in younger populations. However, all these are limited by recruitment to cross-sectional studies, and for this reason, there are some missing data that could affect the results.

## Conclusions

Data reviewed in this work suggest that IFG-associated cognitive deficit could be caused by an increase in oxidation levels coupled with a decrease in antioxidant defense mechanisms. The imbalance between oxidative distress and antioxidant systems increases the phosphorylation of tyrosine kinases, such as focal adhesion kinase, and proteins involved in endothelial cells junction which is associated with increased stress fiber formation and neutrophil-endothelial cell adhesion (Shi et al., 2022). Altogether these changes would conduce to increase BBB permeability and hence increase oxidation levels in the brain, as has been demonstrated *in vitro* in several cell culture models (Figure 1B). Furthermore, the data reviewed here suggest that the cognitive deficit associated with DBT2 starts at the very early stages of the disease, under IFG conditions, before IR development (Figure 1B). This IFG-associated cognitive impairment could be detected in studies comparing young patients with IFG and control volunteers, as the results obtained in cross-sectional studies carried out in older populations did not detect significant differences between these two groups. This leads us to hypothesize that age-dependent cognitive decline may mask/obscure the slight differences in cognitive parameters between IFG and control groups, leading to misinterpretation of the data.

## Author Contributions

MC, KN, and MB performed the bibliographic search. MB and MC wrote the manuscript. MB and KN performed the figure. All authors contributed to the article and approved the submitted version.

## Conflict of Interest

The authors declare that the research was conducted in the absence of any commercial or financial relationships that could be construed as a potential conflict of interest.

## Publisher’s Note

All claims expressed in this article are solely those of the authors and do not necessarily represent those of their affiliated organizations, or those of the publisher, the editors and the reviewers. Any product that may be evaluated in this article, or claim that may be made by its manufacturer, is not guaranteed or endorsed by the publisher.
